# In search of relevance: European political scientists and the public sphere in critical times

**DOI:** 10.1057/s41304-021-00335-9

**Published:** 2021-06-10

**Authors:** José Real-Dato, Luca Verzichelli

**Affiliations:** 1 Department of Law, University of Almería (Spain), Carretera de Sacramento, s/n La Cañada de San Urbano, 04120 Almería, Spain; 2 Department of Social, Political and Cognitive Sciences, University of Siena, Via Mattioli 10, 53100 Siena, Italy

**Keywords:** Political science, Social relevance, Europe, Partisanship, Visibility, Impact, Crisis

## Abstract

Social relevance has become a key element to assess the social legitimacy of an academic discipline. This contrasts with a widespread sentiment among political scientists about the existence of a relevance gap. The context of multiple crises Europe has experienced since the late 2000s has provided political scientists with a multitude of opportunities to demonstrate the social relevance of their work and the usefulness of the discipline. This introductory article to the special issue aims to offer an explorative framework and a preliminary discussion of empirical examples to assess the phenomenon of political scientists’ relevance in the public sphere during recent turbulent times. The framework (which emphasises three basic dimensions of social relevance – partisanship, visibility, and impact) is used to interpret the main results of the five case studies included in the special issue. Results show that contextual factors (salience of the issue, political and media contexts) influence political scientists’ engagement in the public sphere, the role they adopt and their visibility. The article ends emphasising the importance of collective action within the discipline as an instrument to enhance its social relevance.

## Introduction: the debate on political scientists’ relevance during difficult times

How relevant is political science for society? This has been a recurrent question during the last few decades for all scholars interested in the evolution of the discipline (Ricci 1984; Schram and Caterino 2006; Holmberg and Rothstein 2012; Flinders 2013; John 2013; Rogowski 2013; Stoker et al. 2015a). On the one hand, the importance of such a question derives from the intimate relationship between relevance—defined as the ability of political science to be broadly perceived as a valuable instrument for positive social, political or institutional change (Flinders 2013)—and the social legitimacy of the discipline. Though the discipline’s legitimacy may not rely only on instrumental criteria, we must admit that, nowadays, justifying the social value of political science for its own sake has become increasingly difficult (for a more detailed review of this debate, see Monroe 2005; Flinders 2013; John 2013). And, with this concern about the external legitimacy of the discipline in mind, we must also admit that European political scientists do not seem to have a strong influence in their own political systems. The extent of the social and economic usefulness—either in the form of relevance or impact—of political science is somewhat limited, which to some extent explains why political scientists do not heed political scientists (Rogowski 2013). At the same time, relevance has already become a criterion for research funding in many countries (Dunlop 2018; Bandola-Gill et al. 2021). The scenarios opened by the 2020 COVID-19 pandemic will surely amplify the nexus between relevance and resources. Therefore, in a context of increased competition for resources with more established neighbouring disciplines, the real challenge for political scientists will consist in developing their capability to convince the decision makers and the general public about the social usefulness of the discipline. 

On the other hand, the discipline’s relevance is also important from an internal point of view. First, it sets a normative benchmark at the core of the evaluation system, distributing rewards within the community. In this respect, we must not forget that, though politicians and government bureaucrats have, in the last few decades, increased their role in the evaluation of science, implementation of research assessment still largely relies on peer review (Ziman 2000). Therefore, the members of the discipline define, to a large extent, what relevant political science is and what it is not. Moreover, this makes relevance an issue prone to producing conflicts within the discipline, as Stoker et al. point out (2015b, 4). An example of such a situation was the *perestroika debate* in the 2000s (Schram and Caterino 2006),^1^ which clearly shows how contested the concept of relevance is, and how deeply it influences the very identity of the discipline. Second, producing socially relevant political science is not just a way to contribute to the institutional consolidation of the discipline in terms of resources. Indeed, it also constitutes a source of self-esteem for political science academics and practitioners, since it makes them feel more relevant in real life. This recalls what Stoker et al. argue about the interest in relevance among many political scientists as being equivalent to a ‘mid-life crisis’ (2015b, 3): something they worry about when their career is more or less established. However, as the external pressures for relevance and impact increase, we cannot discard that such ‘mid-life crises’ may become a generalised syndrome, involving younger political scientists in their early-stage careers.^2^

Concerning the current situation, there is widespread sentiment among political scientists about the existence of a relevance gap (Ricci 1984; Schram and Caterino 2006; Desch 2019; but see Laitin 2003; John 2013).^3^ A broad corroboration of such sentiment comes from the online survey produced in the context of the PROSEPS project carried out in 2018 among academic political scientists in 35 European countries (plus Israel and Turkey) (Verzichelli et al. 2019).^4^ The survey showed that 79.8 per cent of respondents considered that political scientists have little or no impact on public opinion compared to other academics and public intellectuals.^5^ In addition, 69 per cent of political scientists in Europe saw their involvement with political and social actors participating in policy-making as a normative obligation, while 92 per cent thought they should engage in public debate as part of their role as social scientists.

This introductory article to the special issue aims to offer an explorative framework and a preliminary discussion of empirical examples to assess the phenomenon of political scientists’ relevance in these recent turbulent times. The next section will describe the historical context—basically the first two decades of the twenty-first century. The central section will then present the theoretical framework in which we propose to analyze political scientists’ overall relevance in the public sphere during turbulent times. We will then present the main findings from the case studies included in the issue. All these contributions, although quite diverse in terms of topics covered and methods employed, aim to analyse some recent experiences of public debate engagement of the academic political scientists who have struggled for relevance outside the ivory tower. As Ricci (1984, 300) pointed out, ‘whatever special knowledge or insight political scientists acquire can be maximally useful only if transmitted to society as an intelligible element in the sort of debate that free men have always conducted on public affairs.’ To our knowledge, we still lack studies analysing what concretely happens when political scientists engage in public debates: for instance, the roles they adopt, the content of their discourses, how they deal with normative conflicts, or how the political, social, and media context affects them. The contributions included in this special issue aim to fill this gap.

## European political scientists and the public sphere in times of crisis

There is no doubt that recent years, and the 2010s in particular, have provided opportunities for political science to come down from its ‘ivory tower’ and show how relevant the discipline could be. It has been a difficult period for democratic governance, particularly for the European democracies. First, several major global crises have hit the continent severely. While European societies strove against the grip of the financial and economic crisis in the first half of the decade, the ‘Arab Spring’ (Brownlee et al. 2015) altered geopolitical equilibria in the Southern and Eastern Mediterranean area, spawning political instability in Europe’s southern neighbourhood. One of the outcomes of these events, the Syrian civil war, triggered the most massive migration of history (Sadiki 2015; Mainwaring 2019), with millions of people heading for Europe, searching for a future that had suddenly vanished in their countries of origin. The Syrian war also nurtured the Islamic terrorist threat, which hit several European countries.

Extraordinary events within a few national contexts also characterised the decade, determining substantial repercussions at the supranational level. These include the national independence crises in Scotland and Catalonia, the Greek bailout referendum and the 2016 Brexit referendum. Also, several critical elections (for instance the British elections in 2010 and 2015, the French elections in 2012 and 2017, the Italian elections of 2013 and 2018) led to a deep reconfiguration of party systems and governments all over Europe. In addition, some Central European countries and some European neighbouring countries (Turkey, Israel), challenged the standards of liberal democracy by adopting new policies on immigration and civil rights. Finally, the rise of nationalism and populism and the crisis of legitimacy of traditional actors have also endangered the long-term equilibrium in many European polities and, to some extent, in European Union governance.

These critical events had severe consequences, including mass protests, peaks of polarization and changes in the political compositions of some governments. Some of them affected the inner nature and integrity of national or supranational polities (the Catalan crisis in Spain and Brexit for the European Union). Other events brought internal or external shocks for political systems that ultimately led to significant political reconfigurations. Therefore, it is no surprise that these crises have been a major stimulus for political science research during the last decade, particularly in Europe. Indeed, dozens of books and hundreds of journal articles have appeared during the last few years on the effects of the economic and migration crises on the many dimensions of EU and national political systems.^6^ More in general, notions like ‘democratic disease’ and ‘turbulent times’ are recurrent in several recent political science works, such as those concerned with the crisis of liberal democracy (Mair 2013; Urbinati 2014; Tormey 2015; Howe 2017; Mounk 2018), and those on the growing mistrust of political elites and the rise of populism (Rosanvallon 2008, 2020).

Apart from all this food for academic research, the context of ‘multiple crises’ has also provided political scientists with a multitude of opportunities to demonstrate the social relevance of their work and the usefulness of the discipline. In this perspective, the crucial political significance of many recently debated issues (for instance, the challenges posed by multilevel and global governance, the crisis of liberal democracy, the rise of populist parties and some important constitutional and policy reforms) has motivated many political scientists to descend from their ivory tower willingly, not only to act as informed commentators or advisors. Indeed, they have been induced to talk to decision-makers and be proactive in the public debate.

In this special issue, we review a few recent examples of political scientists' engagement in the public debate. We do not intend to produce a representative sample of all the different situations in which political scientists may become involved in the public sphere. However, the articles cover various political events and contexts, from largely debated systemic crises to country-focused issues of only limited interest to the public. Such a variety of contexts can help us shed some light on the factors that might affect political scientists’ efforts to make their (usually modest) contribution to achieving what they believe would be a better society.

## Political scientists and the public sphere: an interpretative framework

What makes political scientists more inclined to take a proactive role in public debate? In particular, what helps them play such a role when the ‘game gets tough’ due to the growing demands of reforms or the sudden rise of critical challenges for their democratic polity? To answer these questions, we develop a comprehensive framework that we will try to apply to several examples of recent problematic debates about the present and future of European politics.

Our point of departure is the observation about the extent and intensity of political scientists’ presence in public debates. The PROSEPS survey results confirm that participation in the public domain is not uncommon among political scientists: 57.5 per cent of the respondents to the survey admitted having participated in public debates in the media over the preceding three years.^7^ However, the intensity of participation is unevenly distributed, with only a tiny minority (hardly 21 per cent) appearing as highly active.^8^ Hence, not all political scientists seem to have the same predisposition to engage in public debates. This structural pattern primarily relates to individual preferences—some might prefer to devote their time exclusively to what is considered their professional duty (teaching or doing research), while others might feel more attracted to getting involved in the public sphere, either because they feel it is a normative obligation or for the sake of personal notoriety.

Also, contextual or external factors are essential in conditioning political scientists’ public engagement. First, the degree of openness of existing media to the opinions of academics influences the presence of political scientists in the public sphere. The media system's political characteristics (Blumler and Gurevitch 1995; Hallin and Mancini 2004) could also influence the content and approach of political scientists’ interventions, promoting the consolidation of different roles (partisan, broker, or observer).

Second, political circumstances may also influence political scientists’ participation in the public debate. Illiberal political contexts where media exposure may endanger academics’ professional status are not the most appropriate breeding ground for public engagement. Of course, this is just a conditioning factor since participation would depend on each individual's decision to assess the risks of engagement, normative commitments, and the social or political values at stake. The importance of the stakes for the political community (that is, to what extent the issues under discussion affect fundamental political structures and values) and the degree of political polarization of the debate are also external factors conditioning public participation. In this respect, we assume that the rise of peculiar challenges and crises affecting key social values and dealing with political scientists' epistemic expertise may determine a momentum for proactive behaviours among these scholars.

**Fig. 1:**
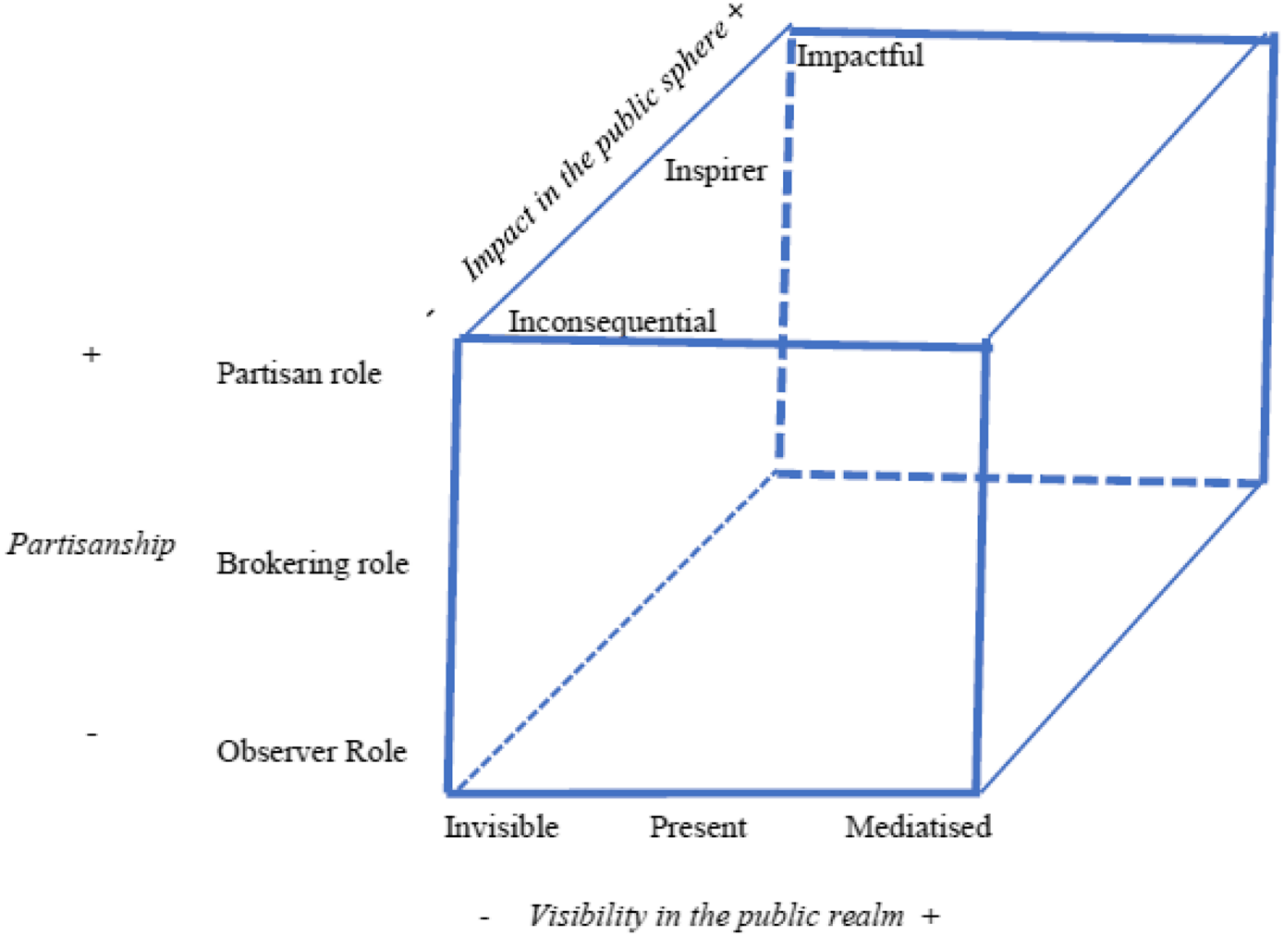
The engagement of political scientists in times of crisis. An interpretative framework

However, here another point of discussion arises concerning the purpose of public engagement. The events discussed in this special issue show the plurality of roles political scientists may play when deciding to participate in the public sphere. We describe these roles by looking to the extent of political scientists' partisanship, which constitutes the first crucial dimension we frame of the space of political science relevance (Fig. 1). Ideally, we distinguish between: a *partisan role,* where interventions imply support for specific political or policy positions; a *brokering role*, where the participants keep a neutral stance but intervene in the political debate by proposing solutions or alternatives to solve a problem that implies some degree of conflict, and an *observer role*, where political scientists limit their interventions to enlighten the public about specific aspects of the issue, without taking sides.

Therefore, for many political scientists, coming down from the ivory tower implies direct involvement in political disputes, equating public engagement with political action. Concerning the public relevance of the discipline, this may have two opposing consequences. On the one hand, the arguments of academic political scientists may become more attractive for those political actors who can use them to legitimate and argumentatively reinforce their positions, especially when the issue is highly salient. Besides, the involvement of scholars and public intellectuals may depend on the level of media politicization (Hallin and Mancini 2004). Indeed, highly politicized media might prefer partisan contributions, while a more neutral approach may be preferred in those contexts where conflicts are already mediated and somehow mitigated by consociational institutions and practices of political bargaining.

On the other hand, partisanship might be a double-edged sword for the relevance of political science. Adopting a partisan stance can be considered a legitimate option whenever it is compatible with a scrupulous separation between scientific inference and political judgement. However, these demanding requirements are not always compatible with providing relevant arguments for political confrontation. Therefore, there is a risk that the struggle for political relevance implies prioritizing political spinning over respect for scientific standards. Actually, this constitutes a danger for the legitimacy and long-term relevance of the discipline. If what political scientists say is perceived just as argumentative ammunition in the political conflict, political science's chances of constructively contributing to the political process or informing evidence-based policy-making could be drastically reduced. This ‘kick back’ effect of the search for the political relevance of political science probably lies at the heart of many scholars' reservations towards an activist conception of social science (i.e. the *perestroika* debate (Monroe 2005)). Moreover, the same effect also shapes their view of relevance as a long-term process inextricably associated with the primary task of contributing to the discipline's scientific advance (John 2013; Rogowski 2013).

The second dimension of our relevance framework is that of visibility, here conceived as the extent of the familiarity of public opinion with political scientists’ work. In this respect, we may distinguish between: a low level of visibility (when scholars are *invisible* to the broad public); a medium level (when they are *present* in the media but they have no protagonist role in public debates as ‘experts’ in a given field), and a high level, when a scholar’s expertise is both recognized by the audience and the media and granted a privileged position in public debates within the current media system. This *mediatization* of political scientists has traditionally occurred in the form of a regular column or a frequent presence in traditional media debates, though nowadays it is complemented with recurrent and highly visible activities in social media.

The third dimension in our scheme is the category of impact, which we define as the ability to influence policy-makers’ decisions (John 2013). In this dimension, political scientists can be: *inconsequential; inspirational* (when they feed policy-makers’ ideas without being directly acknowledged), or *impactful* (when their contribution is effectively recognised). Achieving such recognition is not an easy task. Political science has to compete in the public sphere with more socially established and populated neighbouring disciplines—law, economics, psychology, and even sociology. The latter are better equipped to deal with the output-oriented character of technical policy issues compared with political science and its more process-oriented focus (Scharpf 1997, p. 10–11). This is why the terrain where political scientists are more competitive in the public sphere is, precisely, that of political processes and values, commenting on elections, organizational and institutional dynamics (in government, parliament or parties), and public opinion dynamics. However, even on this terrain, other professional skills (for instance, journalists) may be more effective than political scientists. In any case, despite this competition, the appreciation of the knowledge produced by political scientists in these topics may induce greater access to policy-makers.

Of course, this is just a scheme that probably oversimplifies the compound game of relationships between the visibility, relevance, and impact of political science and probably does not entirely capture the overall ability of a discipline to produce public goods (Flinders 2013). We conceived this framework as a first tool to provide an illustrative comparison of different examples of political scientists’ engagement. Therefore, our first goal is it to check when and how the different opportunities for political scientists' engagement that have emerged in these turbulent times—different types of crises and different national and contextual factors—have changed political scientists' placement along the three dimensions.

We expect that the three dimensions are connected but relatively independent. For instance, gaining *visibility* may be related to political scientists adopting more *partisan* positions or leading to increased impact, though the connection is not necessary in both cases. Besides, though impact requires some visibility, visibility itself does not guarantee impact. Moreover, correlations are expected to be influenced by specific contextual factors such as those mentioned above. Thus, we might expect that the increasing political polarization surrounding a highly salient issue in a politicized media system would make political scientists holding partisan positions more attractive to mass media, increasing the likelihood of becoming more visible to the public. In the same vein, a hostile political context for academics (where they see their professional positions endangered by direct involvement in contentious public debates) might reduce the overall visibility of political scientists in the public sphere. Indeed, these conditions may convince them to give up any kind of participation or to adopt a low, non-partisan profile in those public debates.

Table 1, where we summarise some findings from the articles included in the special issue, reports the different contextual factors at work in the few case studies covered. The first two columns refer to the significance of the stakes for the political community (that is, to what extent the debate affects basic political agreements, values, or structures) and the degree of political polarization of the political debates. The table also indicates the general political context surrounding the debates, that is, the presence of elements conditioning public interventions by political scientists. Basically, we distinguish here those cases where illiberal strains or pressures challenge the basic principles of liberal democracy. Finally, the table indicates the nature (domestic and/or supranational) of the issue at stake.

**Table 1:** Cases in the special issue and variables of interest

	Issue salience	Issue polarization	Political context	Nature of the issues debated	Visibility of P.S	Partisanship of P.S. (predominant role)	Impact of P.S
Bailout referendum in Greece	High	High	Liberal	Supranational	Mediatic (minority)/Invisible (majority)	Partisan	Inconsequential
Constitutional referendum in Italy	High	High	Liberal	Domestic	Mediatic (minority)/Invisible (majority)	Partisan	Inconsequential
Catalonia independence crisis in Spain	High	High	Liberal	Domestic	Mediatic (minority)/Present (some) /Invisible (most)	Partisan	Inconsequential (with exceptions)
Migration crisis in Hungary	High	High	Illiberal	Domestic	Present (minority)/Invisible (majority)	Mixed (Partisan pro-government and observer)	Inconsequential
Several cases of public debates in Finland (Ukraine crisis, EU asylum crisis, and regional government and health reforms)	Low/Medium	Low/medium	Liberal	Mixed	Present (minority)/Invisible (majority)	Observer	Inconsequential
Civic education debate in Israel	Medium	High	Liberal (under strain)	Domestic	Mediatic (minority) invisible (majority)	Partisan	Impactful

See text for the definition of the different levels of each variable

## Main findings from the case studies

Table 1 reports the main results of our case studies. A considerable degree of variability emerges across the cases considered in the articles. The only exception is polarization, which was a common feature in almost all the debates. More specifically, four cases focus on highly salient and significant events occurring during the past decade: the 2015 Greek referendum on the acceptance of the bailout agreement (Tsirbas and Zirganou-Kazolea 2021); the 2016 referendum on the reform of the Italian constitution (Pritoni and Vicentini 2021); and the independence crisis in Catalonia between 2010 and 2018 (Real-Dato et al. 2021). The articles on Finland, Hungary and Israel focus on some cases of public engagement of political scientists in specific policy-related questions. Despite the fact these latter debates attracted a significant degree of attention, they did not affect basic elements in the political structures of the respective countries.

Therefore, the article on Finland (Koikkalainen 2021) covers three issues: the Russian-Ukrainian 2014 crisis, the reform of regional government concerning social and healthcare policies in 2015, and the EU’s asylum crisis in 2015. The article on Hungary (Farkas 2021) also focuses on the 2015 refugee crisis, looking at the attitudes of social scientists towards the divisive policies put in action by the Orbán government. Finally, the article on Israel (Neubauer-Shani 2021) focuses on the participation of political scientists in the design of the civic studies curriculum. Two of these cases are set in countries (Hungary and Israel) where the principles of liberal democracy have been brought into question by government actions in recent years.

The final columns of Table 1 summarize the main features of the involvement of political scientists in the analysed public debates concerning their partisanship, visibility, and impact. Those features are coded according to the different degree of intensity attributed to each of the three variables in the general framework. Therefore, a measure of visibility of political scientists may be coded as more or less evident (distinguishing between majority and minorities of political scientists when applicable). The measure of partisanship can be coded in a continuum between a clearly “partisan” position and a position of pure “observer”. Finally, the work of a given political scientist can be coded along a continuum between “impactful” and “inconsequential”. Overall, the sketchy characterization of the cases presented in Table 1 shows how context may affect political scientists' visibility in the public sphere and the roles they play in public debates. We observe that where the debates dealt with highly polarizing issues affecting basic elements of the political architecture of the respective countries, as in the cases of Greece, Italy, and Spain, political scientists predominantly adopt a partisan stance and are substantially more visible. The latter would probably be as a result of the increased partisanship present in the politically polarized context, as the media would be more receptive to partisan interventions, particularly in those media systems with high levels of politicization, such as Greece, Italy or Spain (Hallin and Mancini 2004; Büchel et al. 2016).

The connection between high political significance, political polarization and a high degree of partisanship of political scientists also applies to the case of Israel. Despite what we could infer from the apparently minor importance of this issue compared with the critical events analysed elsewhere, the civic studies debate was politically important in Israel since, as Michal Neubauer-Shani argues in her article, it reflected a more in-depth debate existing in Israel between politically liberal and conservative views of the nature of its democratic regime and the tensions between its Jewish and liberal components. This divide would take root among members of the Israeli political science community, where a small group of political scientists became highly vocal and visible about the issue.

The issues analysed in the article on Finland seem to reinforce, from a different perspective, the argument about the relationship between political significance and polarization, partisanship, and visibility. Thus, political scientists were not particularly visible in the three issues analysed in the country (Russian-Ukrainian crisis, reform of regional social and healthcare policies, EU’s asylum crisis), and they mostly adopt a brokering approach in their interventions. The nature of these issues and the context might have contributed to this approach. Compared to the referendum debates in Greece or Italy, or to the Catalan independence crisis, the three issues analysed in the article on Finland involved a lower degree of political conflict and polarization. Although the immigration crisis involved some degree of polarization—basically associated with the positions of one of the parties in government—Finnish political forces have shown a substantial degree of agreement on the Russian/Ukrainian conflict and regional and social reforms. In this political context, with a scarcely politicized media system (Nord 2008), political scientists find fewer incentives to adopt a partisan role, preferring to contribute in a more constructive (though probably less visible) fashion.

The case of Hungary also demonstrates the importance of the political context, though as a stimulus for low visibility. In this country, since 2015, immigration from outside the European Union, despite its actual low incidence, became an issue dominating public debate and occupying the centre of the government’s political strategy, which even declared a state of emergency and called for a referendum on immigrant quotas (Várnagy 2017). However, despite the considerable political significance of the issue, political scientists hardly intervened in the public debate. As Eszter Farkas demonstrates in her article, this kind of behaviour is not exclusive to the immigration issue, but it has become generalised among Hungarian political scientists in the last few years. The reason, Farkas argues, lies in the illiberal developments in Hungarian politics since 2010 and, particularly, the capture of most media (public and private) by the government and its allies, and a hostile environment for academic freedom, which has provoked a flight of political scientists from the public sphere. Moreover, the minority of political scientists participating in the debate was who has intervened in this debate was divided between a highly partisan sector supporting government positions and those adopting a purely observer, not politically involved, stance.

Finally, concerning the dimension of the policy-making relevance of public interventions, it is clear from Table 1 that political science scholars have had a rather limited impact in most cases. The only significant exception is the debate in Israel, where a small group of political scientists have made a significant contribution to the configuration of the civic studies policy. Furthermore, the recent events in Italy and Greece show that the outcome of three referenda contradicted the predominant positions of political scientists in the media. Therefore, increased levels of visibility and recognition in the public debate do not automatically lead to an enhanced influence of political scientists, even when the debates are directly related to their sphere of competences.

## The future relevance of political science: opportunities and pitfalls

The case studies briefly summarized above tell us somewhat different stories and illuminate all the difficulties of the discipline in coping with real-world problems and assuming a visible and relevant role in the public sphere. In addition, a somehow perceptible political science ‘voice’, although feeble and not always choral, emerges in specific moments of political turbulence. The ‘lessons’ we can draw from the articles of the special issue enable us to reflect on the opportunities and the problems that engagement in public debates may pose to political scientists.

The studies confirm that European political scientists may have changing predispositions to engage in public debates. Individual preferences due to different role perceptions—teaching duties or ‘publish or perish’ obsessions—are the main reasons behind the disengagement of many, while the idea of engagement as a normative obligation may be frustrated by high levels of political polarization. Providing appropriate incentives has been a commonplace in the discussion among political scientists about the need to foster an appetite for relevance (Stoker 2010; Savage 2013). We extrapolate this by looking to the promotion of participation in the public debate, with measures such as enhancing the consideration of public engagement as a valuable activity in competitions for academic positions or research funding. Some countries have already resorted to these instruments (Dunlop 2018). However, the general picture captured by the case studies and the PROSEPS survey^9^ is that there is still much room for a closer connection between public engagement and career advancement.

The articles in the special issue also remark on the deep gulf existing between public visibility and impact. In line with the different views about political science relevance, some recipes aim to fill this gap. Thus, some stress the claim for the introduction of institutional incentives to promote positive public engagement or policy impact. Others emphasise the importance of laying out relevant questions and answering them with methodological rigour (John 2013), improving the way we communicate research results to stakeholders and the public (Flinders 2013), or focusing on political or policy design as research topics (Stoker 2010). However, the article on Israel (the only one in this collection that provides a positive instance of political scientists' impact in the policy-making process) suggests the importance of collective action. After all, we must not forget that relevance is a collective goal for the discipline and that, therefore, it also requires a collective effort from its members.

Such an effort would imply two fronts of activity. On the internal front, those scholars who believe in the importance of social relevance for the discipline’s institutional development should try to persuade those who are still not aware of it by incentivizing individual and collective behaviours. Here, there is still much work to do, since only a minority (according to the PROSEPS survey) consider that the social impact of research outputs (44.8 per cent) or social and media visibility (27.4 per cent) should become more important requirements for career advancement (PROSEPS 2019). Moreover, on the external front, the profession should collectively struggle to raise awareness about its relevance before the public and potential users, such as governments, public administrations, international organizations, political parties, think tanks, interest groups, or the media.

The most suitable instruments for materializing such internal and external efforts are professional associations, either at the national or international level. Above all, professional associations are fora, where members of the political science community can deliberate and exchange ideas about how the profession should evolve. Besides, they may employ a few tools—conferences, seminars, journals, newsletters, courses, funding schemes—in order to promote the idea of the importance of relevance within the professional community. Finally, professional associations should also act as the collective representatives of the discipline towards external actors and the public, promoting the profession's interests. However, we feel there is still much to do in this respect. First, there is no diffuse concern within the profession that raising public awareness about the discipline’s relevance should be a central mission of political science associations.^10^ Second, due to the scarcity of resources controlled by political science associations in many countries, the capacity to engage in comprehensive actions to stimulate political science’s relevance is much lower than other academic ‘guilds’, like historians or lawyers.

In a nutshell, climbing down from the ivory tower is never easy. Even more so, it is not easy for a still young, vulnerable, polymorphic academic discipline formed of several types of individual competencies but a very tiny minority of eclectic observers. It is probably not a tragedy, compared to the tragedies of the ‘real world’. But, this may certainly be a long-term problem for European political science.

## Footnotes

1 The *Perestroika* movement emerged within the US community of political scientists to oppose the hegemony of quantitative and formalistic methodology and to support the idea the discipline should produce a body of more comprehensible and (therefore) more relevant pieces of research. On the roots of this movement see also Monroe (2005).

2 In this respect, about a third of those PhD students surveyed by Savage (2013, 197) considered engaging with non-academic actors as one of the top-three priorities in their training period.

3 A review of the variety of positions in this debate can be found in the book edited by Stoker et al. (2015a) and the special issue of *Political Studies Review* that originated it.

4 The target population of the survey was identified by national experts in each country, either using official domestic definitions of membership in the discipline or, when these were not available, the basic criteria of: a) either having a PhD in political science or working in political science units in universities or research centres; and b) most of their research activity or teaching assignments dealing with political science topics. A total of 12,442 individuals were included in the final survey population. The fieldwork took place between March and December 2018. The total number of complete responses gathered was 2,354, The average response rate by country was 26 per cent, ranging from 7 per cent in Turkey or 10 per cent in UK, to 58 per cent in Italy.

5 Political scientists in Denmark, Iceland, and Sweden were the only exceptions to this generalised feeling.

6 A bibliographic search in the Social Science Citation Index (SSCI) database of articles including the terms ‘crisis’ and ‘Euro*’ in the keywords, abstract or the title, and published in political science journals between 2010 and 2020, produced a total of 2034 results. Also, 1676 articles included one of these expressions: ‘Eurozone crisis’, ‘financial crisis’ or ‘economic crisis’. In turn, the number of articles including the term ‘Brexit’ in the title, keywords or abstract, amounted to 534 (Web of Science, https://apps.webofknowledge.com/, accessed 26 September 2020).

7 This figure comes from respondents’ self-reporting. This is, however, the only information we have about the general patterns of participation in the public debate of political scientists. Given the possibility of biases that self-selection issues could introduce into the data (which we believe should not be different from those found in any other similar opinion survey using self-reporting), these data should be taken as illustrative rather than at face value.

8 This group is formed by those respondents who self-reported that they had appeared in one of the three traditional media (newspapers, radio and TV) at least once a month and/or participated in public debate through digital media (Twitter, Facebook, blogs) at least once a week.

9 On a 0–10 scale, where ‘0’ means that ‘participation of political scientists is not recognised at all’ and ‘10’ that ‘it is fully recognised’, the average score for the whole sample (N = 2304) is 4.46. Across countries, this average is above ‘5’, only in 11 out of 39 countries with the highest scores corresponding to Latvia (6.38) and Lithuania (6.28). However, only 45% of interviewees think that impact should become a more important requirement for career advancement, and a much lower percentage (28%) think the same of public visibility.

10 Only a minority (46.6 per cent) of the respondents to the PROSEPS survey considered that ‘promoting a stronger presence of political science in the public sphere’ should be one of the three main missions of national political science associations. This was, however, the second item over which respondents showed greater agreement, only below the goal of ‘promoting academic debate and exchange’ (57.8 per cent), and far ahead of the third and fourth most mentioned missions: ‘offering guidance and professional training to young generations of scholars’ and ‘protecting/defending the professional interests of their members’ (31.5 and 30.2 per cent) (PROSEPS, 2019).
